# Burnout and Sleep Quality in Nurses in the COVID-19 Pandemic

**DOI:** 10.4314/ejhs.v35i3.7

**Published:** 2025-05

**Authors:** Sevil Olğun, Derya Adibelli

**Affiliations:** 1 Aydın Adnan Menderes University, Nursing Faculty, Department of Fundamental of Nursing, Aydın, Turkey; 2 Antalya Bilim University Health Science Faculty, Department of Nursing, Antalya, Turkey

**Keywords:** Burnout, Health, Nurses, Pandemic, Sleep quality

## Abstract

**Background:**

This study aimed to examine nurses' burnout and sleep quality during the COVID-19 pandemic.

**Methods:**

A cross-sectional design was employed. Data were collected from 256 nurses working in COVID-19 units between July 2021 and January 2022 using the Nurse Identification Form, the Maslach Burnout Scale (MBS), and the Pittsburgh Sleep Quality Index (PSQI).

**Results:**

Participants had worked in COVID-19 units for an average of 11.41 ± 9.11 months, with 50.6% employed in state hospitals. Significant differences were observed in the emotional exhaustion and depersonalization subscale scores of the MBS based on the type of COVID-19 unit and nurses' work patterns (p < 0.05). A significant positive correlation was found between PSQI scores and emotional exhaustion (p < 0.05).

**Conclusion:**

Nurses who experienced higher emotional exhaustion during the pandemic also reported poorer sleep quality.

## Introduction

Coronavirus Disease 2019 (COVID-19) has significantly impacted individuals and the labor force globally, primarily due to its high mortality and morbidity rates. Healthcare professionals particularly nurses have borne the brunt of this crisis (1–3). Nurses were at greater risk due to their close and prolonged contact with COVID-19 patients (4, 5). As a result, the pandemic disrupted nurses' working conditions, family lives, social relationships, physical and mental health, and, notably, sleep patterns (2, 4, 6).

Reports indicate a rise in sleep disturbances, with both duration and quality declining due to demanding schedules and constant exposure to the virus (4, 7, 8). Studies have documented reduced sleep quality (6, 9), increased stress (4, 8), and elevated feelings of burnout among nurses (9, 10). Sleep deprivation and fatigue among nurses can undermine patient safety by impairing job performance, increasing workplace accidents, and affecting cognitive functions and clinical decision-making (4). Globally, burnout and sleep disturbances among nurses have worsened with the pandemic (11, 12).

Understanding the extent of burnout and sleep issues among nurses during the pandemic can help inform better workforce policies and support systems. This study was conducted to evaluate burnout levels and sleep quality in nurses and to explore the relationship between the two during the COVID-19 pandemic.

## Methods

**Setting and sample**: This cross-sectional study was conducted between July 2021 and January 2022 among nurses working in COVID-19 wards, intensive care units (ICUs), and follow-up/treatment units at state and university hospitals, as well as in primary healthcare services (PHCS) in a province in western Türkiye. A post-hoc power analysis using G*Power 3.1.9.4 revealed that the sample size of 256 provided 81% power at a 95% confidence level with an effect size of 0.31. Inclusion criteria were nurses who had worked for at least one month in COVID-19-related departments.

**Data collection tools**: Data were gathered via the Nurse Identification Form, Maslach Burnout Scale (MBS), and Pittsburgh Sleep Quality Index (PSQI). The Nurse Identification Form included questions about demographic characteristics and sleep habits (1, 11).

The MBS is a 22-item scale measuring emotional exhaustion, depersonalization, and personal accomplishment using a 5-point Likert scale. Higher scores in emotional exhaustion and depersonalization and lower scores in personal accomplishment indicate higher burnout (13). Cronbach's alpha values were 0.91 for emotional exhaustion, 0.74 for depersonalization, and 0.75 for personal accomplishment (12).

The PSQI assesses sleep quality over the previous month through 19 self-rated items divided into seven components. Scores range from 0 to 21; scores >5 indicate poor sleep quality (14). The scale's Cronbach's alpha was 0.80 (15). Surveys were distributed and collected via WhatsApp over six months. Completion took approximately 15–20 minutes.

**Data analysis**: SPSS version 25.0 was used. Descriptive statistics, IQR, skewness, kurtosis, Mann–Whitney U test, t-test, ANOVA, Kruskal–Wallis H test, Tukey's post hoc test, and correlation analyses were performed.

**Ethical considerations**: Ethical approval was obtained from the university ethics committee (Decision No: 2021/253). Institutional permissions were secured. Participants gave electronic informed consent.

## Results

The average age of participants was 32.10 ± 7.92 years. They had worked as nurses for 10.40 ± 8.40 years and in COVID-19 units for 11.41 ± 9.11 months. Average weekly working hours increased from 40.0 (8.0) before the pandemic to 48.0 (16.0) during it. The median number of patients cared for daily was 8.0 (13.0), and the number of monthly night shifts was 6.0 (9.0). Daily sleep duration decreased from 8.0 (1.0) hours pre-pandemic to 6.0 (2.0) hours during the pandemic; post-night shift sleep dropped to 4.21 ± 2.36 hours.

Emotional exhaustion and depersonalization scores were significantly higher among nurses in pandemic wards, ICUs, and outpatient clinics (p < 0.05). Personal accomplishment scores were lower among ICU and charge nurses (p < 0.05). Night shift workers and those on rotating shifts had significantly higher emotional exhaustion and depersonalization scores (p < 0.05) (Table 1).

**Table 1:** Comparison of participants' characteristics according to Maslach Burnout Scale and subscale mean scores (n=256)

	Emotional	Depersonalisation	Personal Success
Variables	Exhaustion		
	Mean±SD	Mean±SD	Mean±SD
**Gender**			
Female	23.19±8.77	7.89±5.06	10.54±5.94
Male	22.89±8.05	9.10±5.57	8.42±4.08
**Statistics**	t=0.142	t=-0.968	t=1.987
	p=0.887	p=0.335	p=0.056
**Marital status**			
Married	22.38±8.94	7.46±5.03	10.51±6.54
Single	24.00±8.32	8.65±5.18	10.02±5.27
**Statistics**	t=-1.166	t=-1.447	t=0.530
	p=0.155	p=0.150	p=0.597
**Health institution where he/she is employed**			
State hospital	22.37±8.90	7.59±4.72	9.62±6.07
University hospital	24.32±8.33	8.23±5.42	10.65±5.44
Primary health care services	22.15±8.82	9.76±5.86	12.46±5.25
**Statistics**	F=0.989	F=1.085	F=1.587
	p=0.374	p=0.341	p=0.208
**Department of employment**			
Pandemic outpatient clinic^1^	16.11±7.83	5.35±4.49	10.94±7.12
Pandemic unit^2^	23.78±9.51	8.75±5.50	11.89±5.55
Pandemic ward^3^	23.78±7.42	7.16±4.42	9.83±5.80
COVID-19 intensive care unit^4^	24.33±7.41	8.96±5.25	9.41±5.40
**Statistics**	**F=4.538**	**F=2.968**	F=1.580 p=0.197
	**p=0.004**	**p=0.034**	
	**2,3,4>1**	**2,3,4>1**	
**Position in the department of employment**			
Clinical/intensive care nurse^1^	24.52±7.95	8.54±5.00	9.62±5.61
Supervisor nurse^2^	15.86±7.91	6.06±4.51	8.26±5.21
Outpatient clinic/unit nurse^3^	22.76±9.20	7.65±5.45	12.23±5.87
**Statistics**	**F=7.030**	F=1.709	**F=4.374**
	**p=0.001**	p=0.185	**p=0.014**
	**1,3>2**		**3>1,2**
**Working order**			
Continuous daytime^1^	18.62±10.00	6.42±5.56	11.11±6.78
Continuous night^2^	29.00±5.44	13.66±6.37	11.50±6.77
Daytime/night variable shift system^3^	24.23±7.85	8.23±4.71	9.96±5.41
**Statistics**	F=7.636	F=5.762	F=0.665 p=0.516
	**p=0.001**	**p=0.004**	
	**2,3>1**	**2>1,3**	

p<0.05, t: independent groups t-test; F: one-way analysis of variance

Single nurses had significantly higher sleep latency scores than married nurses (p < 0.05). Habitual sleep efficiency was higher in outpatient clinic and rotating-shift nurses. Daytime dysfunction scores were higher among ward nurses and those with ICU responsibilities. Subjective sleep quality scores were higher among ICU nurses and continuous night-shift workers (p < 0.05). Overall, PSQI scores were significantly higher among single nurses and those working in pandemic wards, ICUs, and rotating shifts (p < 0.05) (Table 2).

**Table 2:** Comparison of participants' characteristics according to Pittsburgh Sleep Quality Index mean scores (n=256)

Characteristics	n	Mean±SD	Statistics
**Gender**			
Female	137	11.40±3.36	t=-0.275
Male	19	11.63±3.75	p=0.784
**Marital status**			
Married	81	10.90±3.46	t=-2.033
Single	75	12.00±3.27	p=0.044
**Health institution where he/she is employed**			
State hospital	79	11.81±3.46	F=1.008
University hospital	64	11.01±3.17	
Primary health care services	13	11.15±4.09	p=0.367
**Department of employment**			
Pandemic outpatient clinic^1^	17	10.94±3.47	F=3.845
Pandemic unit^2^	37	12.09±3.30	p=0.011
Pandemic ward^3^	42	11.90±3.08	3,4>1,2
COVID-19 intensive care unit^4^	60	10.94±3.47	
**Position in the department of employment**			
Clinical/intensive care nurse^1^	94	11.90±3.04	F=2.635 p=0.075
Supervisor nurse^2^	15	10.13±3.70	
Outpatient clinic/unit nurse^3^	47	10.89±3.85	
**Working order**			
Continuous daytime^1^	35	9.82±3.85	F=5.604
Continuous night^2^	6	10.83±3.65	p=0.004
Daytime and night variable shift system^3^	115	11.94±3.11	3>1,2

p<0.05, IQR: interquartile range; t: independent groups t-test; F: one-way analysis of variance; U: Mann-Whitney U test; KW: Kruskal-Wallis test

A moderate positive correlation was found between emotional exhaustion and sleep disturbance, and between emotional exhaustion and sleep duration. Depersonalization showed a weak positive correlation with both. Emotional exhaustion also had a weak positive correlation with subjective sleep quality. Use of sleeping medication was weakly positively correlated with personal accomplishment. Total PSQI scores were moderately positively correlated with emotional exhaustion (p < 0.05) (Table 3).

**Table 3:** Correlation of the Pittsburgh Sleep Quality Index and Maslach Burnout Scale subdimensions scores (n = 256)

		1	2	3	4	5	6	7	8	9	10	11
Sleep latency	*r*	-										
	*p*	-										
Habitual sleep activity	*r*	.196	-									
	*p*	**.014**	-									
Sleep disturbance	*r*	.376	.115	-								
	*p*	**.000**	.153	-								
Daytime dysfunction	*r*	.243	.312	.331	-							
	*p*	**.002**	**.000**	**.000**	-							
Sleep duration	*r*	.324	.391	.349	.336	-						
	*p*	**.000**	**.000**	**.000**	**.000**	-						
Subjective sleep quality	*r*	.358	.177	.392	.395	.335	-					
	*p*	**.000**	**.028**	**.000**	**.000**	**.000**	-					
Use of sleeping pills	*r*	.078	.211	.274	.125	.257	.251	.506				
	*p*	.173	**.000**	**.000**	**.028**	**.000**	.082	**.000**				
PSQI total	*r*	.657	.594	.572	.627	.721	.473	.617	-			
	*p*	**.000**	**.000**	**.000**	**.000**	**.000**	.095	**.000**	-			
Emotional exhaustion	*r*	.144	.182	.316	.378	.172	.020	.366	.362	-		
	*p*	.073	**.023**	**.000**	**.000**	**.032**	.808	**.000**	**.000**	-		
Depersonalisation	*r*	.124	.064	.183	.167	.083	.151	.233	.226	.647	-	
	*p*	.123	.424	**.023**	**.037**	.304	.061	.003	.004	**.000**	-	
Personal success	*r*	-	-	.004	.019	-	.226	-	-	.176	.284	-
		.032	.052			.018		.081	.030			
	*p*	**.691**	**.522**	**.963**	**.810**	**.819**	**.005**	**.316**	**.712**	**.028**	**.000**	-

PSQI: Pittsburgh Sleep Quality Index

## Discussion

The COVID-19 pandemic placed extraordinary psychological and physical demands on nurses, increasing their risk of burnout (16). Emotional exhaustion and depersonalization were most pronounced among ICU and ward nurses, corroborating prior studies (20–22). ICU nurses were especially vulnerable due to high patient loads, resource shortages, and fear of infection (16, 20).

Rotating and continuous night shifts contributed to elevated burnout scores. This aligns with findings by Temur et al. (5) and others (17, 23), who reported higher emotional exhaustion and depersonalization among night-shift and rotating-shift nurses.

Sleep quality was also significantly affected. Nurses in COVID-19 ICUs and wards, and those working rotating shifts, had poorer sleep quality, as shown by elevated PSQI scores. Single nurses also reported longer sleep latency and lower sleep quality, though some studies did not find significant marital status differences (1, 7).

Work-related stressors such as excessive workloads, high infection risk, and PPE challenges likely contributed to poor sleep quality (4, 24, 25). Consistent with this, nurses working night or rotating shifts exhibited higher PSQI scores. These findings mirror earlier research on shift work and sleep disruption among healthcare workers (24–26).

Finally, the study found a significant correlation between burnout and poor sleep quality, echoing previous studies (1, 5, 27). Nurses experiencing higher emotional exhaustion reported greater sleep disturbance. Pandemic-related stressors including fear of death, uncertainty, isolation, and resource constraints likely exacerbated both burnout and sleep problems.

The inclusion of nurses from diverse healthcare settings enhances the study's generalizability. Conducting the study during the pandemic, with nurses actively treating COVID-19 patients, adds relevance and objectivity. However, the cross-sectional design limits causal inferences. Online data collection may have introduced selection bias, favoring tech-savvy nurses and excluding those not active on social media.

This study highlights significant emotional exhaustion and poor sleep quality among nurses during the COVID-19 pandemic, particularly those working in ICUs and on night shifts. A clear inverse relationship was observed between burnout and sleep quality. To mitigate these issues, healthcare systems should consider rotating ICU assignments, implementing equitable shift schedules, and offering psychological support. Improvements in working conditions, increased staffing, and pandemic-specific policies are also essential to safeguard nurses' well-being.

## References

[1] Aydin Sayilan A, Kulakaç N, Uzun S (2021). Burnout levels and sleep quality of COVID-19 heroes. Perspect Psychiatr Care.

[2] Huang L, Lei W, Liu H (2020). Nurses' sleep quality of “fangcang” hospital in china during the COVID-19 pandemic. International Journal of Mental Health and Addiction.

[3] Stojanov J, Malobabic M, Stanojevic G (2021). Quality of sleep and health-related quality of life among health care professionals treating patients with Coronavirus disease-19. International Journal of Social Psychiatry.

[4] Salari N, Khazaie H, Hosseinian-Far A (2020). The prevalence of sleep disturbances among physicians and nurses facing the COVID-19 patients: a systematic review and metaanalysis. Globalization and Health.

[5] Temur H, Turan N, Aştı T (2023). Sleep quality and burnout levels in nurses during the COVID-19 pandemic process: descriptive and relationship seeking study. Turkiye Klinikleri Journal of Nursing Sciences.

[6] Xiao H, Zhang Y, Kong D, Li S, Yang N (2020). The effects of social support on sleep quality of medical staff treating patients with Coronavirus disease-2019 (COVID-19) in January and February in China. Med Sci Monit.

[7] Demirtürk Selçuk E, Demirağ BC (2021). The effect of COVID-19 outbreak on secondary traumatic stress and sleep quality in healthcare workers. Turkiye Klinikleri J Nurs Sci.

[8] Şahin MK, Aker S, Şahin G, Karabekiroğlu A (2020). Prevalence of depression, anxiety, distress and insomnia and related factors in healthcare workers during COVID-19 pandemic in Turkey. J Community Health.

[9] Cecere L, de Novellis S, Gravante A (2023). Quality of life of critical care nurses and impact on anxiety, depression, stress, burnout and sleep quality: A cross-sectional study. Intensive and Critical Care Nursing.

[10] Yılmaz S, Düşükcan M (2022). Covid-19 pandemi sürecinde hemşirelerde tükenmişlik ve kaygının yaşam kalitesi üzerine etkisi. Hacettepe Sağlık İdaresi Dergisi.

[11] Feng HL, Qi XX, Xia CL, Xiao SQ, Fan L (2021). Association between night shift and sleep quality and health among Chinese nurses: A cross-sectional study. J Nurs Manag.

[12] Orsal O, Orsal O, Duru P, Unsal A, Barlas N (2017). Evaluation of the factors associated with Burnout of nurses working at a state hospital in turkey. Nursing Practice Today.

[13] Ergin C, Bayraktar R, Dağ İ (1992). Doktor ve hemşirelerde Tükenmişlik Maslach Tükenmişlik Ölçeği'nin uyarlanması. VII. Ulusal Psikoloji Kongresi Bilimsel Çalışmaları.

[14] Buysse DI, Reynolds CR, Monk TH, Berman SR, Kupfer DI (1989). The Pittsburgh Sleep Quality Index: A new instrument for practice and research. Psychiatry Research.

[15] Ağargün MY, Kara H, Anlar O (1996). Pittsburgh uyku kalitesi indeksi'nin geçerliği ve güvenirliği. Türk Psikiyatri Dergisi.

[16] Toscano F, Tommasi F, Giusino D (2022). Burnout in intensive care nurses during the COVID-19 pandemic: a scoping review on its prevalence and risk and protective factors. Int. J. Environ. Res. Public Health.

[17] Edis EK, Keten M (2022). COVID-19 pandemi sürecinde sağlık çalışanlarında tükenmişlik, iş tatmini ve yaşam doyumu. Kırıkkale Üniversitesi Tıp Fakültesi Dergisi.

[18] Yılmaz M, Kıraç Y, Sahin MK (2021). Sleep quality and related factors in a sample of Turkish healthcare workers during the COVID-19 pandemic: a cross-sectional study. International Journal of Clinical Practice.

[19] Bahia Galal A (2022). Hassan Siam, Latifah Nawaf Alrasheedi. Burnout among emergency nurses during COVID-19 pandemic at Hail Governmental Hospitals in the Kingdom of Saudi Arabia. Ethiop J Health Sci.

[20] Bruyneel A, Smith P, Tack J, Pirson M (2021). Prevalence of burnout risk and factors associated with burnout risk among ICU nurses during the COVID-19 outbreak in French speaking Belgium. Intensive and Critical Care Nursing.

[21] Solmaz A, Kutlu A (2023). Determination of the relationship between levels of compassion fatigue and burnout in nurses during the covid-19 outbreak. Celal Bayar University-Health Sciences Institute Journal.

[22] Uysal-Toraman A, Kısa Ö (2023). The burnout levels of nurses working in covid-19 ward and intensive care units and associated factors: A cross-sectional study. Cyprus Turkish Journal of Psychiatry & Psychology.

[23] Hoşgör DG, Tanyel TÇ, Cin S, Demirsoy SB (2021). COVID-19 pandemisi döneminde sağlık çalışanlarında tükenmişlik: İstanbul ili örneği. Avrasya Sosyal ve Ekonomi Araştırmaları Dergisi.

[24] Kirhan I, Uzer F (2021). Evaluation of sleep quality and daytime sleepiness in nurses. Annals of Medical Research.

[25] Bhat S, Chokroverty S (2022). Sleep disorders and COVID-19. Sleep Medicine.

[26] Tarhan M, Aydin A, Ersoy E, Dalar L (2018). The sleep quality of nurses and its influencing factors. Eurasian J Pulmonol.

[27] Doustinouri M, Rahnama M, Abdollahimohammad A (2024). Effect of positive thinking skills on optimism and death anxiety of COVID-19 nurses: A quasi-experimental study. Ethiopian Journal of Health Sciences.

